# Analysis of prognostic factors for survival after curative resections of clear-cell renal cell carcinoma based on Bayesian networks

**DOI:** 10.3389/fonc.2026.1877632

**Published:** 2026-10-28

**Authors:** Bin Zhan, Yachun Chen, Hongqian Ke, Sixing Huang, Siwei Xu, Yi Huang, Xiaobao Chen, Xuru Zhang, Yuxin Wu, Wei Jiang, Ronghui Lin, Tianqi Lin, Shushang Chen

**Affiliations:** 1 Department of Urology, Clinical Oncology School of Fujian Medical University, Fujian Cancer Hospital, Fuzhou, China; 2 Department of Infectious Disease, The First Affiliated Hospital of Fujian Medical University, Fuzhou, China; 3 Department of Gynecology, Clinical Oncology School of Fujian Medical University, Fujian Cancer Hospital, Fuzhou, China; 4 Department of Urology, Zhangzhou Affiliated Hospital of Fujian Medical University, Zhangzhou, China; 5 Department of Urology, The Second Affiliated Hospital of Fujian Medical University, Quanzhou, China; 6 Department of Urology, Fujian Medical University Union Hospital, Fuzhou, China; 7 Department of Anesthesiology, Clinical Oncology School of Fujian Medical University, Fujian Cancer Hospital, Fuzhou, China; 8 Department of Radiology, Clinical Oncology School of Fujian Medical University, Fujian Cancer Hospital, Fuzhou, China

**Keywords:** Bayesian network, clear cell renal cell carcinoma, disease-free survival, machine learning, overall survival, prognostic modeling

## Abstract

**Background:**

Accurate risk stratification following curative resection of non-metastatic clear cell renal cell carcinoma (nm-ccRCC) remains clinically challenging. This study aims to develop and validate Bayesian network (BN) models to predict survival in patients with nm-ccRCC.

**Methods:**

A dual-center retrospective cohort was randomly stratified into a training set (N = 326) and an internal validation set (N = 140). External validation was performed using an independent external cohort (N = 170). BN models were constructed based on clinicopathologic characteristics that demonstrated statistical significance in univariate analyses of the training cohort and were assessed using receiver operating characteristic (ROC) curves, confusion matrices, calibration and decision curve analysis.

**Results:**

The Bayesian analyses identified T stages as the principal determinant of 5-year overall survival (OS), while ISUP grades emerged as the dominant predictor for 3-year disease-free survival (DFS). The 5-year OS and 3-year DFS models achieved areas under the ROC curve (AUCs) of 0.878 and 0.867 in the internal validation cohort and 0.838 and 0.846 in the external validation cohort. Calibration slopes were close to unity with small intercepts across cohorts, and decision curve analysis showed net benefit exceeding that of treating all or no patients across a wide range of thresholds.

**Conclusions:**

This study establishes BNs for the first time as a clinically actionable tool for postoperative risk prediction in nm-ccRCC, with promising discriminative and calibrated performance supporting their potential use in patient counseling, risk-stratified surveillance, and the selection of candidates for adjuvant therapy or clinical trials. Future prospective, multicenter studies are warranted to extend these predictions to a 5-year DFS endpoint and to refine the decision threshold for clinical translation.

## Introduction

According to Global Cancer Statistics 2022, there are more than 434,000 new cases of renal cancer and more than 155,000 deaths worldwide each year (1). In recent years, due to the increased availability of abdominal ultrasound and cross-sectional imaging examinations, most patients present with early-stage tumors at the time of presentation. For localized renal cell carcinoma (RCC), partial nephrectomy (PN) and radical nephrectomy (RN) are the standard curative treatments. However, after curative resection of non-metastatic RCC, as many as 20%-40% of patients still experience recurrence or metastasis (2–5). Clear cell renal cell carcinoma (ccRCC), which accounts for 75-85% of RCC, is the most common type of renal malignancy in adults (6, 7). Currently, for high-risk patients with non-metastatic clear cell renal cell carcinoma (nm-ccRCC) after curative resections, pembrolizumab therapy and clinical trial participation constitute viable therapeutic options. The KEYNOTE-564 study has demonstrated that adjuvant pembrolizumab significantly improves overall survival (OS) and disease-free survival (DFS) in patients with ccRCC who are at increased risk of recurrence (8, 9). Hence, the early identification of patients predisposed to adverse prognosis following curative resection, coupled with the implementation of intensified surveillance, adjuvant therapies, or clinical trial enrollment, remains of paramount clinical importance. Aiming at the differential prognosis of patients with nm-ccRCC, previous studies have developed a variety of prediction models, such as the University of California Integrated Staging System (UISS) (10), the Leibovich score (11), and the GRANT score (12). However, most prognostic models oversimplify by using limited parameters, neglecting multifactorial interplay, and often excluding partial nephrectomy cohorts. Furthermore, when validated in external and prospective cohorts, these models uniformly demonstrated unsatisfactory predictive efficacy (13). Therefore, developing more comprehensive, precise, and up-to-date prognostic models to guide postoperative decision-making remains a significant challenge in nm-ccRCC research.

The concept of Bayesian networks (BNs), initially introduced by Pearl in 1986 (14), represents a probabilistic graphical model characterized by a directed acyclic graph coupled with a conditional probability table. Unlike previous models that primarily relied on multivariate Cox regression analyses and nomograms, BNs offer a dual advantage in modeling complex interrelationships among variables. First, they provide an intuitive representation of intricate probabilistic dependencies through network diagrams. Second, BNs facilitate the inference of posterior probabilities for various events by adjusting prior probabilities and conditional probability distributions, thereby serving as valuable tools in decision-making processes. Recently, BNs have gradually garnered increasing attention in the analytical fields of malignancies such as endometrial cancer, gallbladder carcinoma, and lung cancer (15–17). BN-based analyses can extract effective information that is unknown in advance but has potential predictive value, thus providing a scientific foundation for the comprehensive identification of risk factors and the accurate prediction of disease risks. In this study, we constructed and validated precise BN models to forecast DFS and OS after curative surgery in nm-ccRCC patients, aiming to equip surgeons with dependable information for customizing treatment strategies. To our knowledge, this is the first report employing BNs to predict postoperative survival in patients with nm-ccRCC.

## Materials and methods

### Patients and data collection

Patients with sporadic nm-ccRCC who underwent curative resections at Fujian Medical University Union Hospital, Zhangzhou Affiliated Hospital of Fujian Medical University, and Fujian Cancer Hospital from January 2016 to June 2019 were included. Exclusion criteria comprised: 1. Metastatic disease (including extra-regional lymph node metastasis); 2. Postoperative tumor remnants or positive margins; 3. Concurrent malignancies; 4. Bilateral or hereditary RCC; 5. Incomplete clinical or follow-up data; 6. Postoperative adjuvant therapy. Clinicopathologic characteristics collected included: age at the time of surgery, current or previous smoking history, sex, diabetes, hypertension, heart diseases, symptoms, body mass index (BMI), Eastern Cooperative Oncology Group Performance Status (ECOG-PS), preoperative serum albumin, preoperative estimated glomerular filtration rate (eGFR), International Society of Urological Pathology (ISUP) grades, type of surgery, laterality, tumor necrosis, microvascular invasion, maximal tumor diameter, T stages, and N stages. T stages were determined from surgical pathology. N stages were classified based on radiographic evidence and/or histopathological confirmation. Tumor necrosis referred to the presence of coagulative necrosis observed under the microscope. Microvascular invasion was identified by the infiltration of tumor cells into intratumoral microvessels or the presence of tumor emboli within these microvessels. Follow-up data were obtained through outpatient visits, mailed questionnaires, and telephone interviews. All patients were followed until death or until July 2024. This anonymous retrospective study was conducted under the approval of the Ethics Committee of Fujian Cancer Hospital with a waiver of informed consent (Approval No. K2025-272-01).

### Statistical analysis

All patients from Fujian Cancer Hospital and Fujian Medical University Union Hospital were randomly assigned to a training cohort (N = 326) and a test cohort (N = 140) in a ratio of 7:3. The external validation cohort was from Zhangzhou Affiliated Hospital of Fujian Medical University (N = 170). Continuous variables were expressed as medians and interquartile ranges (IQR). Differences between categorical variables were analyzed using χ^2^ test. Univariate analyses were conducted using Cox regression models. Survival curves were plotted and compared using the Kaplan-Meier method and log-rank test. Statistical analyses were performed using SPSS 26.0 (IBM Corp., Armonk, NY, USA). P < 0.05 was considered statistically significant.

### Outcome definitions

OS was defined as the interval from the date of curative surgery (radical or partial nephrectomy) to the date of all-cause death, with patients alive at the last follow-up censored at the date of last contact. DFS was defined as the interval from the date of curative surgery to the first documented event, whichever occurred first, among (i) local recurrence confirmed histologically or radiologically, (ii) distant metastasis confirmed by imaging and/or pathology, and (iii) all-cause death without prior documented recurrence. Death without documented recurrence was therefore counted as a DFS event. Recurrence status was ascertained during routine postoperative surveillance and verified against imaging and pathology records during data extraction. The start point for both endpoints was the date of surgery, and patients without any documented event were censored at the date of the last follow-up. No distinction between cancer-specific and non-cancer death was made; all deaths within the follow-up window were counted as events. Two prediction horizons were evaluated. Five-year OS was adopted as the primary endpoint because 5-year survival is the most widely reported and clinically interpretable long-term outcome after curative resection of nm-ccRCC and is the metric reported by the established prognostic tools used here for comparison (UISS (10), the Leibovich score (11), the SSIGN score (18), the GRANT score (12) and the SYSUCC nomogram (19)). Three-year DFS was assessed in parallel because it captures recurrence-related risk, the outcome most directly relevant to postoperative surveillance and to decisions regarding adjuvant therapy. The rationale for this choice of DFS horizon is set out in the Discussion, and the two endpoints were analyzed separately; no composite endpoint was used.

### Construction of BNs

The training cohort was composed of 326 patients for BN modeling. The target variable for prediction was survival outcomes (5-year OS and 3-year DFS). Variables demonstrating significant associations (P < 0.05) in univariate analyses were considered attribute variables affecting the state of the target variables. IBM SPSS Modeler 18 (IBM, Armonk, NY, USA) and Netica 3.2 (Norsys Software Corporation, Vancouver, Canada) were utilized to build BN models based on the Tree-Augmented Naive Bayesian (TAN) algorithm.

### Prior probability and posterior probability

Prior probability represents the initial probability distribution of a hypothesis, derived from either existing biological knowledge or empirical training data. Posterior probability is calculated through Bayesian updating of the prior probability by integrating the likelihood function with the observed evidence. In the training phase, the conditional probability distributions between nodes were calibrated using maximum likelihood estimation on the training cohort data. In the classification phase, the posterior probability was computed via exact inference algorithms considering all observed evidence variables. For individual patients, the posterior probabilities of key outcome variables (e.g., 5-year OS and 3-year DFS) could be dynamically updated as new clinical parameters were incorporated into the model.

### Calibration assessment

Because the models are intended to provide individualized survival probabilities, discrimination alone is not sufficient to establish their predictive value, and calibration was therefore assessed in the training, internal validation and external validation cohorts. Calibration, that is the agreement between predicted probabilities and observed event frequencies, was assessed graphically with calibration plots relating the predicted probability to the observed event proportion, and quantified by the calibration intercept and the calibration slope obtained from logistic regression of the observed outcome on the log-odds of the predicted probability, where an intercept of 0 and a slope of 1 indicate perfect calibration. The Brier score, a measure of overall accuracy that combines discrimination and calibration, was also computed. Calibration was assessed with R version 4.5.3 (R Foundation for Statistical Computing, Vienna, Austria).

### Decision curve analysis

Substantive clinical utility requires not only that a model rank patients correctly but that acting on its predictions improves decisions. Decision curve analysis (DCA) was therefore used to quantify the net benefit of the BN models across a range of threshold probabilities, that is, the probability of the adverse outcome above which an intervention would be considered indicated. For a given threshold probability pt, net benefit was calculated as (true positives/n) - (false positives/n) × [pt/(1 - pt)], where n is the number of patients, and was compared with the net benefit of two default strategies, namely treating all patients and treating none. Because the classification metrics reported below are defined with the event-free state as the positive class, whereas DCA is expressed with respect to the adverse outcome that would prompt an intervention, the predicted probability of the adverse outcome (the complement of the predicted probability of the favorable state) was used as the model output for these analyses. Decision curve analysis was performed for the 5-year OS and 3-year DFS models in the training, internal validation and external validation cohorts using R version 4.5.3 (R Foundation for Statistical Computing, Vienna, Austria).

### Model validation and assessment

The confusion matrices were used to summarize the overall performance of models. Accuracy, sensitivity, specificity, positive predictive value (PPV), negative predictive value (NPV), F1-score, and Matthews correlation coefficient (MCC) were calculated based on the confusion matrix. The F1-score is the harmonic mean of PPV and sensitivity, where a value closer to 1 indicates better model performance. The MCC is particularly suitable for imbalanced datasets, as it comprehensively considers the prediction performance of each category. MCC values range from -1 to 1, where 1 represents a perfect prediction, 0 indicates a random prediction, and -1 corresponds to an exact opposite prediction. In addition, the AUC provides another way to evaluate the predictive efficacy of the BNs.

### Definition and interpretation of the classification metrics

The positive class in the confusion matrix analysis is defined as the event-free (favorable) state: for the 5-year OS model a patient is classified as positive when alive at 5 years after surgery, and for the 3-year DFS model a patient is classified as positive when free of recurrence or death at 3 years after surgery. The negative class therefore denotes the adverse outcome of interest, namely all-cause death within 5 years or recurrence or death within 3 years. All metrics are computed with respect to this coding and must be interpreted accordingly: sensitivity is the proportion of patients who were actually event-free and were correctly classified as event-free; specificity is the proportion of patients who actually experienced the adverse outcome and were correctly identified as having experienced it; PPV is the probability that a patient classified as event-free truly remained event-free; and NPV is the probability that a patient classified as having the adverse outcome truly experienced it. The BN classifier used the maximum posterior probability as its default decision rule, and the metrics reported here therefore characterize the models at this default operating point rather than under a clinically optimized threshold. Re-tuning the decision threshold in order to favor higher sensitivity for the adverse-event class would require an explicit threshold-selection procedure, which was not part of the present analysis. Consistent with this coding, a patient who died within 3 years of surgery without a documented recurrence was classified as negative, because DFS was defined with all-cause death as an event.

## Results

### Population characteristics

A total of 636 eligible nm-ccRCC patients were enrolled between January 2016 and June 2019. Patients’ ages ranged from 18 to 85 years, with a median age of 55 years. The median follow-up duration was 67.0 months (IQR 63.7–70.5). Other baseline characteristics are summarized in **Table 1**. No significant differences were observed in baseline characteristics between the training and test cohorts. Due to regional variations among data centers, the external validation cohort exhibited certain disparities in age and laterality compared to the training cohort. The survival curves are depicted in **Figure 1**. The 5-year OS rates were 88.3% (95% CI 84.9-91.8) in the training cohort, 86.4% (95% CI 80.8-92.1) in the internal validation cohort and 85.9% (95% CI 80.6-91.1) in the external validation cohort, and 87.3% (95% CI 84.7-89.9) in the total study population; the corresponding 3-year DFS rates were 83.7% (95% CI 79.7-87.7), 83.6% (95% CI 77.4-89.7), 80.6% (95% CI 74.6-86.5) and 82.9% (95% CI 79.9-85.8), respectively. Log-rank tests indicated no statistically significant differences in either OS (**Figure 1A**) or DFS (**Figure 1B**) between the training cohort and the test cohort, or between the training cohort and the external validation cohort.

**Table 1:** Clinicopathological characteristics for the training cohort, test cohort, and external validation cohort.

Variable	Total, n (%)	Training cohort, n (%)	Test cohort, n (%)	External validation cohort, n (%)	Training cohort vs. Test cohort, P	Training cohort vs. external validation cohort, P
Age (year)					0.668	0.047
≤50	208 (32.7)	100 (30.7)	40 (28.6)	68 (40.0)		
51-69	360 (56.6)	195 (59.8)	83 (59.3)	82 (48.2)		
≥70	68 (10.7)	31 (9.5)	17 (12.1)	20 (11.8)		
Sex					0.650	0.606
Male	440 (69.2)	226 (69.3)	100 (71.4)	114 (67.1)		
Female	196 (30.8)	100 (30.7)	40 (28.6)	56 (32.9)		
BMI (kg/m²)					0.439	0.675
<18.5	36 (5.7)	15 (4.6)	10 (7.1)	11 (6.5)		
18.5-24.9	407 (64.0)	213 (65.3)	85 (60.7)	109 (64.1)		
≥25	193 (30.3)	98 (30.1)	45 (32.1)	50 (29.4)		
Smoking history					0.310	0.557
No	421 (66.2)	210 (64.4)	97 (69.3)	114 (67.1)		
Yes	215 (33.8)	116 (35.6)	43 (30.7)	56 (32.9)		
Diabetes					0.802	0.597
No	542 (85.2)	280 (85.9)	119 (85.0)	143 (84.1)		
Yes	94 (14.8)	46 (14.1)	21 (15.0)	27 (15.9)		
Hypertension					0.697	0.403
No	460 (72.3)	234 (71.8)	98 (70.0)	128 (75.3)		
Yes	176 (27.7)	92 (28.2)	42 (30.0)	42 (24.7)		
Heart diseases					0.264	0.375
No	600 (94.3)	311 (95.4)	130 (92.9)	159 (93.5)		
Yes	36 (5.7)	15 (4.6)	10 (7.1)	11 (6.5)		
Symptoms					0.301	0.713
No	470 (73.9)	239 (73.3)	109 (77.9)	122 (71.8)		
Yes	166 (26.1)	87 (26.7)	31 (22.1)	48 (28.2)		
ECOG-PS					0.083	0.333
0	464 (73.0)	236 (72.4)	112 (80.0)	116 (68.2)		
≥1	172 (27.0)	90 (27.6)	28 (20.0)	54 (31.8)		
Laterality					0.169	0.037
left	323 (50.8)	152 (46.6)	75 (53.6)	96 (56.5)		
right	313 (49.2)	174 (53.4)	65 (46.4)	74 (43.5)		
Serum albumin					0.917	0.877
< 35 g/L	69 (10.8)	36 (11.0)	15 (10.7)	18 (10.6)		
≥ 35 g/L	567 (89.2)	290 (89.0)	125 (89.3)	152 (89.4)		
eGFR (mL/min/1.73m^2^)					0.996	0.461
< 59	233 (36.6)	115 (35.3)	49 (35.0)	69 (40.6)		
60–89	276 (43.4)	143 (43.9)	62 (44.3)	71 (41.8)		
≥90	127 (20.0)	68 (20.9)	29 (20.7)	30 (17.6)		
ISUP grades					0.533	0.698
1	154 (24.2)	80 (24.5)	36 (25.7)	38 (22.4)		
2	301 (47.3)	146 (44.8)	70 (50.0)	85 (50.0)		
3	151 (23.7)	82 (25.2)	29 (20.7)	40 (23.5)		
4	30 (4.7)	18 (5.5)	5 (3.6)	7 (4.1)		
Type of surgery					0.555	0.608
PN	355 (55.8)	182 (55.8)	74 (52.9)	99 (58.2)		
RN	281 (44.2)	144 (44.2)	66 (47.1)	71 (41.8)		
Tumor necrosis					0.739	0.809
No	566 (89.0)	290 (89.0)	126 (90.0)	150 (88.2)		
Yes	70 (11.0)	36 (11.0)	14 (10.0)	20 (11.8)		
Microvascular invasion					0.944	0.654
No	585 (92.0)	301 (92.3)	129 (92.1)	155 (91.2)		
Yes	51 (8.0)	25 (7.7)	11 (7.9)	15 (8.8)		
T stages					0.531	0.739
T1a	294 (46.2)	149 (45.7)	72 (51.4)	73 (42.9)		
T1b	177 (27.8)	94 (28.8)	37 (26.4)	46 (27.1)		
T2	64 (10.1)	34 (10.4)	13 (9.3)	17 (10.0)		
T3a	71 (11.2)	36 (11.0)	10 (7.1)	25 (14.7)		
T3b-T4	30 (4.7)	13 (4.0)	8 (5.7)	9 (5.3)		
N stages					0.899	0.897
N0	593 (93.2)	304 (93.3)	131 (93.6)	158 (92.9)		
N1	43 (6.8)	22 (6.7)	9 (6.4)	12 (7.1)		
Maximal tumor diameter (cm)					0.190	0.916
≤4	308 (48.4)	152 (46.6)	78 (55.7)	78 (45.9)		
>4, ≤7	224 (35.2)	117 (35.9)	43 (30.7)	64 (37.6)		
>7	104 (16.4)	57 (17.5)	19 (13.6)	28 (16.5)		

BMI, Body Mass Index; ECOG-PS, Eastern Cooperative Oncology Group Performance Status; eGFR, Estimated Glomerular Filtration Rate; PN, Partial nephrectomy; RN, Radical nephrectomy; ISUP, International Society of Urological Pathology.

**Figure 1:**
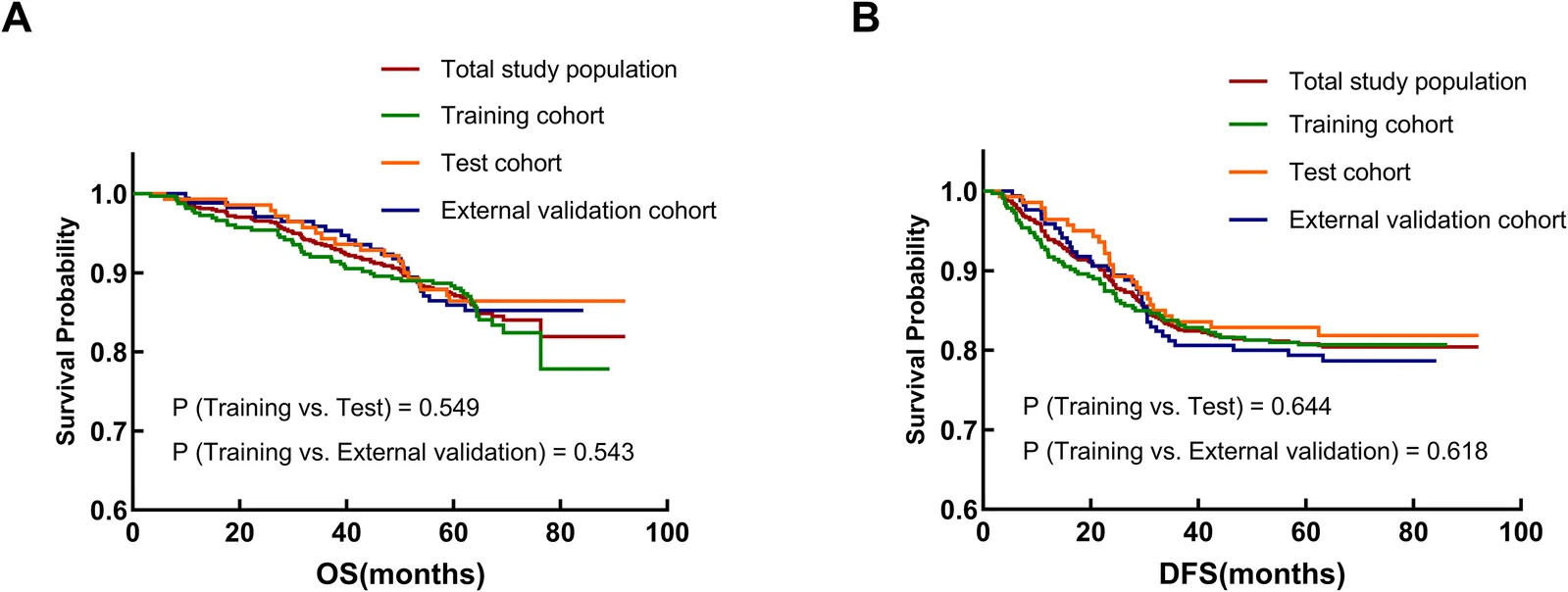
Plots of survival curves. **(A)** Survival curves of OS; **(B)** Survival curves of DFS.

### Model development

Univariate analyses showed that age at the time of surgery, symptoms, ECOG-PS, preoperative eGFR, ISUP grades, type of surgery, tumor necrosis, microvascular invasion, maximal tumor diameter, T stages, and N stages were significantly associated with OS (**Supplementary Table 1**) and DFS (**Supplementary Table 2**). The BNs (**Figures 2A, B**) were constructed using IBM SPSS Modeler 18, with the above variables as inputs and survival outcomes as target variables. It was evident from the BNs predicting 5-year OS and 3-year DFS that age was the parent node of eGFR, indicating that the conditional association of age with survival was conveyed not only by age itself but also by eGFR. Similarly, the maximal tumor diameter was the parent node of the type of surgery, indicating a conditional dependency between tumor size and the choice of surgical approach, both of which were associated with survival outcomes. Notably, age had no parent node in any of the two BNs, indicating that age was conditionally independent of the other covariates in both models. In the 5-year OS model, T-stages showed both a direct conditional association with 5-year OS and additional conditional dependencies conveyed through symptoms, ISUP grades, tumor necrosis, microvascular invasion and maximal tumor diameter. Likewise, T-stages showed additional conditional dependencies with 3-year DFS through symptoms, N-stages, tumor necrosis, microvascular invasion and maximal tumor diameter. The remaining variables can be analyzed in a similar manner.

**Figure 2:**
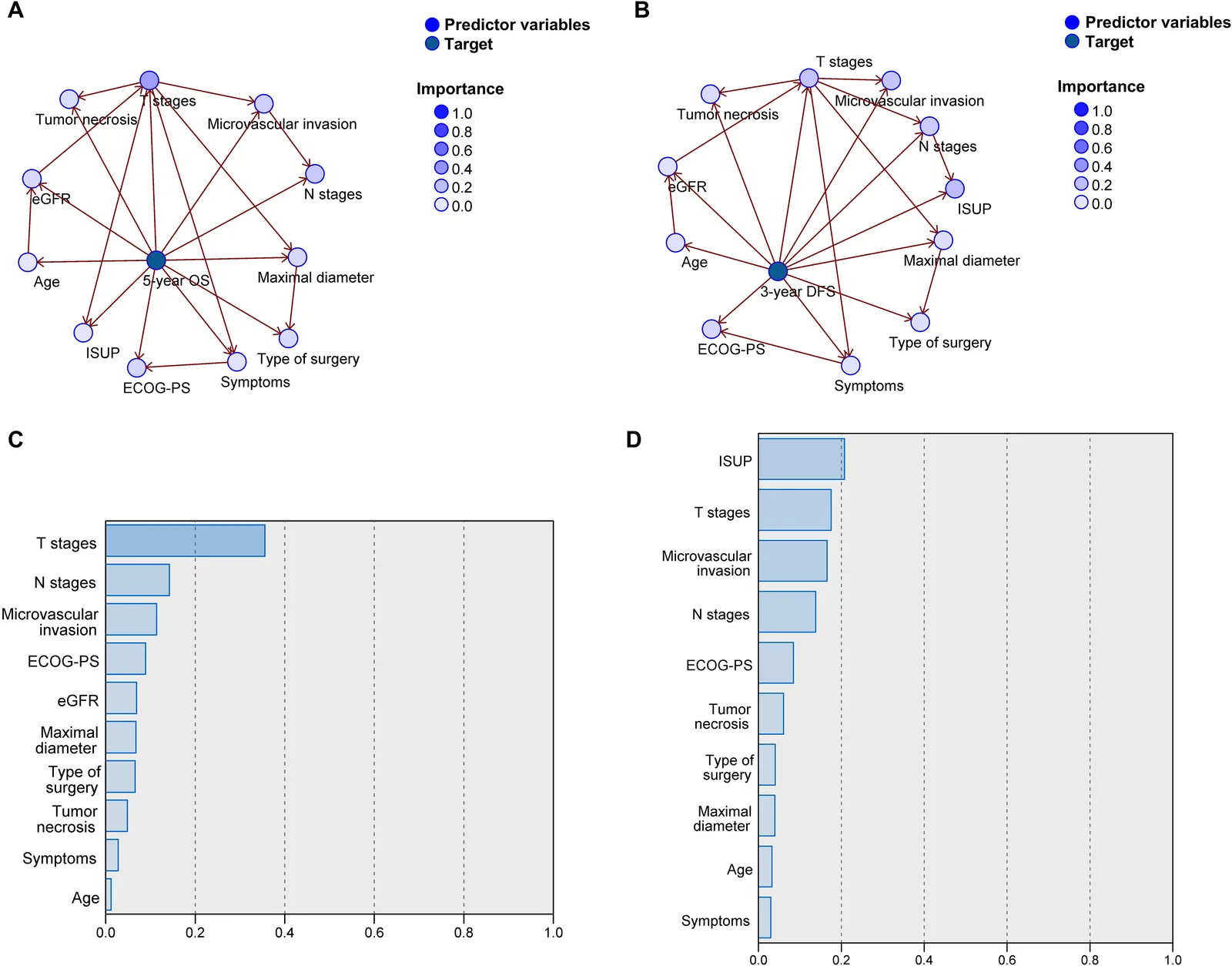
Results of Bayesian analysis using IBM SPSS Modeler 18. **(A)** The TAN BNs predicting 5-year OS; **(B)** The TAN BNs predicting 3-year DFS; **(C)** Importance scores of prognostic factors for 5-year OS; **(D)** Importance scores of prognostic factors for 3-year DFS.

### Importance of prognostic factors

The top ten prognostic factors ranked by importance are shown in **Figures 2C, D**. The results showed that T-stages were the most important factor affecting patients’ 5-year OS. The other four variables in the top five of prognostic importance were N-stages, microvascular invasion, ECOG-PS, and eGFR. Additionally, ISUP grades emerged as the most important prognostic factor affecting patients’ 3-year DFS, accompanied by other significant variables in the following order: T-stages, microvascular invasion, N-stages, and ECOG-PS.

### Conditional probability

The posterior probabilities of T-stages under the upper-level node conditions in the BN model for 5-year OS are shown in **Supplementary Table 3**. The predicted probability of T1a stage was 36% when the patient’s OS was greater than or equal to five years and the eGFR was less than 59 mL/min/1.73 m². **Supplementary Table 4** presents the posterior probabilities of ISUP grades under the upper-level node conditions in the BN model for 3-year DFS. The predicted probability of ISUP grade 4 was 39% when a patient had lymph node metastases and a DFS of less than three years. In a similar manner, the conditional probabilities of other variables can also be inferred.

### Survival prediction

BNs (**Figure 3**) built with Netica 3.2 can provide individualized survival predictions based on the given variable information. For a 72-year-old asymptomatic patient with tumor necrosis, pT3aN0 stage, an ECOG-PS score of 0, an eGFR of 70 mL/min/1.73 m², an ISUP grade of 2, a maximal tumor diameter of 6 cm, and no microvascular invasion, the predicted 5-year OS and 3-year DFS rates following RN were 66.9% (**Figure 3A**) and 51.2% (**Figure 3B**), respectively. Similarly, survival outcomes can be projected for other patients.

**Figure 3:**
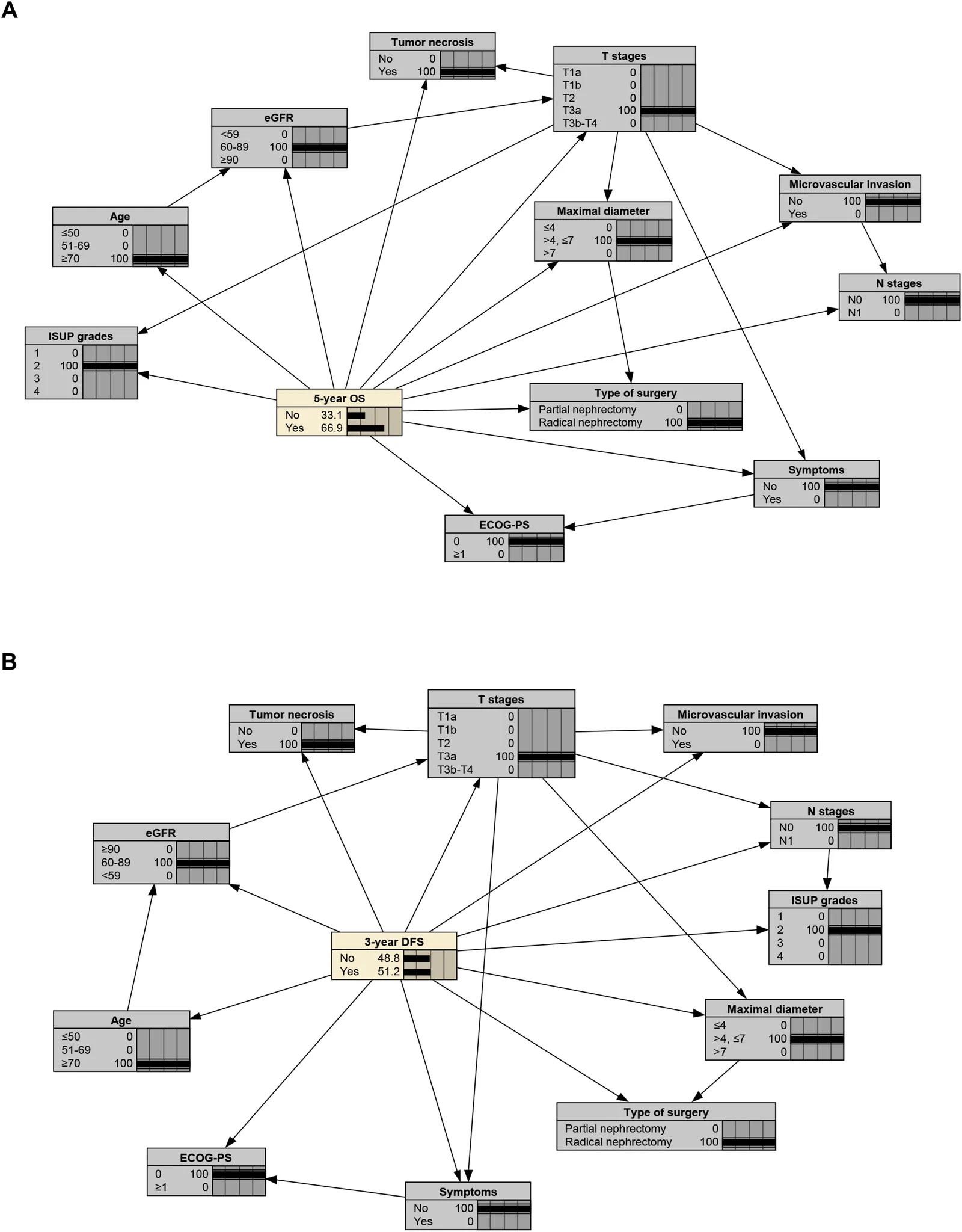
BNs built with Netica 3.2 for a 72-year-old asymptomatic patient with tumor necrosis, pT3aN0 stage, an ECOG-PS score of 0, an eGFR of 70 mL/min/1.73 m², an ISUP grade of 2, a maximal tumor diameter of 6 cm, and no microvascular invasion. **(A)** Survival prediction of 5-year OS; **(B)** Survival prediction of 3-year DFS.

### Validation and assessment of models

The ROC curves of the BN models constructed from the test cohort and external validation cohort are illustrated in **Figure 4**. Compared with the test cohort, the external validation cohort exhibited slight reductions in AUC values, with the 5-year OS model decreasing from 0.878 (**Figure 4A**) to 0.838 (**Figure 4C**) and the 3-year DFS model declining from 0.867 (**Figure 4B**) to 0.846 (**Figure 4D**). As summarized in **Table 2**, the confusion matrix analyses for both internal and external validations indicated sustained high performance in accuracy, sensitivity, PPV, F1-score, and MCC, whereas specificity and NPV were slightly lower.

**Figure 4:**
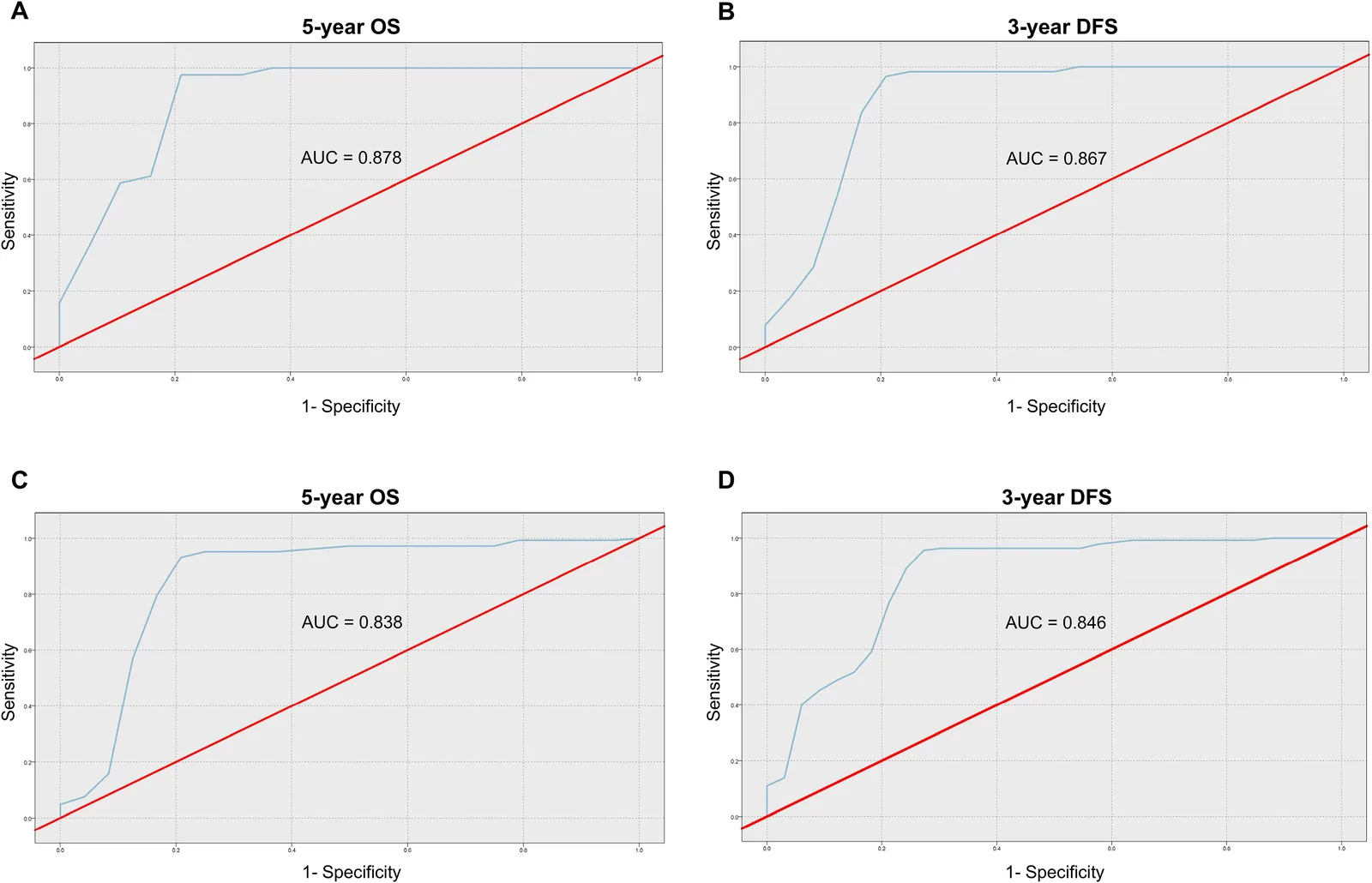
The ROC curves of the BN models. **(A)** The ROC curves of the 5-year OS model in the test cohort; **(B)** The ROC curves of the 3-year DFS model in the test cohort; **(C)** The ROC curves of the 5-year OS model in the external validation cohort; **(D)** The ROC curves of the 3-year DFS model in the external validation cohort.

**Table 2:** The confusion matrix and AUC of the BN models.

Group	Actual	Predicted		Accuracy	Sensitivity	Specificity	PPV	NPV	F1-score	MCC	AUC
		Negative	Positive								
Internal validation (5‐year OS)	Negative	15	4	0.950	0.975	0.789	0.967	0.833	0.971	0.782	0.878
	Positive	3	118								
Internal validation (3‐year DFS)	Negative	19	5	0.943	0.974	0.792	0.958	0.864	0.966	0.798	0.867
	Positive	3	113								
External validation (5‐year OS)	Negative	19	6	0.929	0.959	0.760	0.959	0.760	0.959	0.719	0.838
	Positive	6	139								
External validation (3‐year DFS)	Negative	25	8	0.918	0.956	0.758	0.942	0.806	0.949	0.731	0.846
	Positive	6	131								

Accuracy = (TP + TN)/(TP + FP + TN + FN), Sensitivity=TP/(TP+FN), Specificity=TN/(FP+TN), Positive predictive value(PPV)=TP/(TP+FP), Negative predictive value(NPV)=TN/(FN+TN), F1-score=2×PPV×Sensitivity / (PPV + Sensitivity), Matthews correlation coefficient (MCC) = (TP×TN - FP×FN) / sqrt((TP + FP)×(TP + FN)×(TN + FP)×(TN + FN)), TN true negative, TP true positive, FN false negative, FP false positive. The positive class is the event-free (favourable) state (survival at 5 years after surgery for the OS model; freedom from local recurrence, distant metastasis or death at 3 years after surgery for the DFS model); the negative class denotes the adverse outcome.

### Calibration and decision curve analysis of the BN models

Calibration plots for 5-year OS and 3-year DFS are presented in **Figures 5A, B**, and DCA in **Figures 5C, D**; the complete numerical results, including those for the training cohort, are summarized in **Table 3**. In the internal validation cohort the calibration intercepts were 0.093 (95% CI -0.026 to 0.236) for 5-year OS and 0.143 (95% CI -0.039 to 0.215) for 3-year DFS, with slopes of 0.960 (95% CI 0.852 to 1.071) and 0.956 (95% CI 0.901 to 1.041) and Brier scores of 0.061 (95% CI 0.035 to 0.096) and 0.057 (95% CI 0.028 to 0.091); in the external validation cohort the corresponding values were 0.171 (95% CI -0.011 to 0.281) and 0.161 (95% CI -0.047 to 0.266), with slopes of 0.920 (95% CI 0.789 to 1.056) and 0.946 (95% CI 0.811 to 1.063) and Brier scores of 0.082 (95% CI 0.055 to 0.112) and 0.083 (95% CI 0.052 to 0.114). The slopes were therefore close to unity and the intercepts small in both validation cohorts, indicating close agreement between predicted probabilities and observed event frequencies. Across a wide range of threshold probabilities, DCA showed that the net benefit of using the BN models to guide decisions exceeded that of both treating all patients and treating none, supporting their potential utility for risk-stratified management.

**Figure 5:**
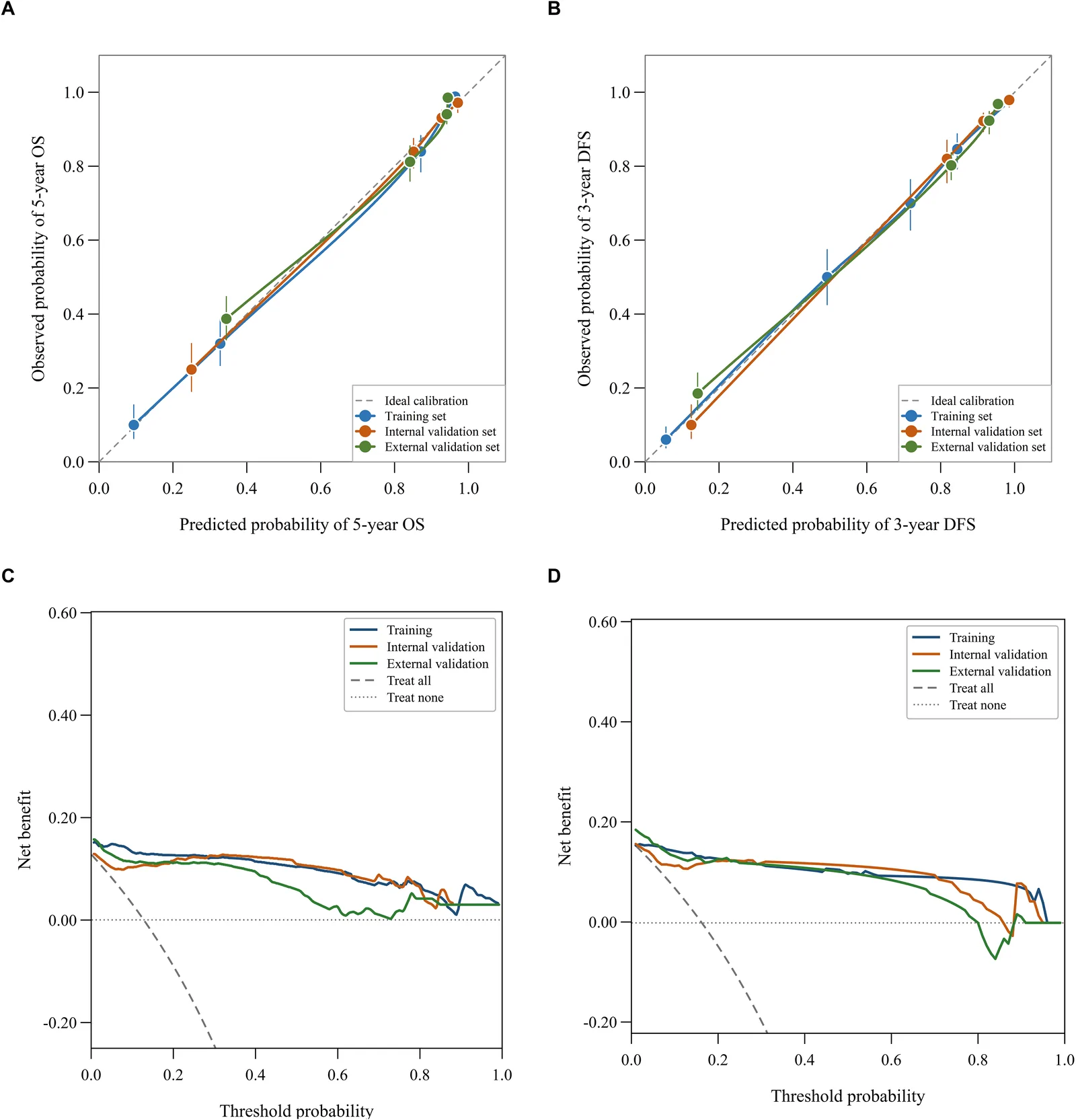
Calibration plots and decision curve analyses of the BN models for 5-year OS and 3-year DFS in the training, internal validation and external validation cohorts. **(A)** Calibration plot of the 5-year OS model; **(B)** Calibration plot of the 3-year DFS model; **(C)** Decision curve analysis of the 5-year OS model; **(D)** Decision curve analysis of the 3-year DFS model.

**Table 3:** Calibration of the BN models for 5-year OS and 3-year DFS in the training, internal validation and external validation cohorts.

Group	Calibration intercept (95% CI)	Calibration slope (95% CI)	Brier score (95% CI)
Training cohort (5‐year OS)	0.052 (−0.092 to 0.121)	1.104 (0.992 to 1.189)	0.041 (0.027 to 0.059)
Internal validation (5‐year OS)	0.093 (−0.026 to 0.236)	0.960 (0.852 to 1.071)	0.061 (0.035 to 0.096)
External validation (5‐year OS)	0.171 (−0.011 to 0.281)	0.920 (0.789 to 1.056)	0.082 (0.055 to 0.112)
Training cohort (3‐year DFS)	0.073 (−0.047 to 0.182)	1.050 (0.952 to 1.157)	0.043 (0.035 to 0.058)
Internal validation (3‐year DFS)	0.143 (−0.039 to 0.215)	0.956 (0.901 to 1.041)	0.057 (0.028 to 0.091)
External validation (3‐year DFS)	0.161 (−0.047 to 0.266)	0.946 (0.811 to 1.063)	0.083 (0.052 to 0.114)

## Discussion

ccRCC represents the most prevalent histological subtype of renal malignancies (20). The selection of adjuvant therapy for patients with nm-ccRCC following curative resection is predominantly contingent upon the risk stratification of tumor recurrence. Prior evidence has substantiated that adjuvant immunotherapy confers survival benefits in patients at high risk of recurrence, underscoring the imperative for precise risk stratification (8, 9). Conventional prognostic models, predominantly derived from multivariate Cox regression analysis, assign prognostic scores based on identified independent risk factors (11, 18, 21, 22). However, this methodology is inherently limited by its potential omission of critical covariates and its failure to model their complex interactions. From a multivariate perspective, the interrelated prognostic determinants of nm-ccRCC are difficult to represent within the additive proportional-hazards framework of Cox regression, in which each covariate contributes independently to the linear predictor unless interactions are specified in advance. Furthermore, the structure of parallel variables in Cox regression analysis lacks the discriminatory power to differentiate between direct risk factors and intermediary factors, which impedes the elucidation of interactive effects among various prognostic determinants in the postoperative progression of nm-ccRCC.

BNs, characterized by their flexibility in accommodating diverse data distributions and their capacity to delineate multi-level variable interdependencies, offer a useful paradigm for investigating latent variables in medical research (17, 23, 24). The Bayesian analytical framework, which integrates principles of probability theory and graph theory, enables both the analysis of prognostic factors and the graphical representation of complex interactions among independent variables and their network relationships with dependent variables. This approach facilitates a comprehensive exploration of conditional dependencies among prognostic variables and the identification of previously unrecognized associations. Moreover, while conventional Cox regression-based prognostic models generally require complete covariate data—a requirement often unattainable in clinical settings—BNs demonstrate a robust capability to handle incomplete data, enabling reliable risk stratification based on partial variable information. The present study employs the BN algorithm to integrate comprehensive clinical, pathological, and radiological parameters with complex interdependencies for postoperative risk prediction in patients with nm-ccRCC. The BN models facilitate real-time predictions of survival outcomes for individual patients, even in scenarios with incomplete data, providing valuable guidance for postoperative therapeutic strategies and follow-up management. By identifying patients with a significantly elevated predicted risk of recurrence, there is a compelling rationale for advocating adjuvant therapy and implementing rigorous follow-up surveillance.

The directed edges learned by the networks encode probabilistic dependencies and conditional independencies among variables; because the structure was learned from observational data without a prespecified causal framework, these edges do not by themselves support a causal or mediating interpretation, and the conditional dependencies reported above should be read as probabilistic rather than causal. Establishing causal relationships would require an explicitly specified causal framework, for example a directed acyclic graph constructed from prior biological knowledge, together with the corresponding identification assumptions, and lies beyond the scope of the present analysis.

External data on the timing of recurrence after curative resection support the choice of a 3-year DFS horizon. The great majority of postoperative recurrences cluster within the first 3 postoperative years. In a prospective single-center series of 187 patients with pT1–3 N0-Nx M0 RCC treated by radical nephrectomy, 80% of all metastases were diagnosed within 3 years of surgery, at a median of 14.5 months (25), and a comprehensive review of adjuvant therapy in RCC similarly reported a median time to relapse of 18 months, with the majority of relapses occurring within 3 years of surgical resection (26). Risk-stratified surgical series are consistent with this pattern, with reported median times to recurrence of 28.9, 17.8 and 9.5 months for low-, intermediate- and high-risk groups, respectively (5). In line with this temporal pattern, current guidance supports concentrating surveillance in the early postoperative period: a synthesis of the American Urological Association and National Comprehensive Cancer Network surveillance protocols concluded that, because approximately half of all recurrences occur during the first 2 years after surgery, “a clear rationale exists for optimizing surveillance strategies after surgery,” while acknowledging the paucity of high-quality evidence (27); the EAU and AUA/NCCN guidelines nonetheless differ substantially in the frequency and type of imaging recommended, and the 2025 European Association of Urology guidelines state that intensive radiological surveillance may not be necessary for all patients, recommending instead a risk-adapted schedule guided by subtype-specific risk models (28). Taken together, these external data indicate that the interval covered by the 3-year DFS endpoint is that in which both recurrence risk and guideline-recommended surveillance intensity are highest, which is the setting in which the model is intended to be used. A second, dataset-specific consideration reinforces this choice. Although the median follow-up of the cohort was 67.0 months (IQR 63.7-70.5), the intensity of cross-sectional imaging surveillance beyond 3 years after surgery was not uniform, and for a proportion of patients who remained alive beyond 3 years progression status could not be reliably ascertained even though vital status was known. A 5-year DFS endpoint would therefore have required assigning an event status to patients whose progression status was unknown, introducing misclassification of unpredictable direction. By contrast, 5-year OS could be retained because vital status was ascertainable for all patients through outpatient review, mailed questionnaires and telephone interviews.

The BN models facilitate the identification of conditional dependency structures among candidate prognostic factors associated with postoperative prognosis in patients with nm-ccRCC. Denic et al. (29) have documented that aging contributes to a decline in glomerular filtration rate at a rate of 6.3 mL/min/1.73 m² per decade. Multiple studies have demonstrated that preoperative baseline glomerular filtration rate significantly influences postoperative renal function and overall prognosis (30–32). Consistent with these results, within the BN models constructed in this study, age was conditionally associated with survival outcomes through its dependency with eGFR and related downstream nodes. Since tumor size serves as a critical determinant in the decision-making process between RN and PN (33–35), our analyses demonstrated that maximal tumor diameter showed a conditional dependency with prognosis that was conveyed through the choice of surgical approach. In addition, symptom status was conditionally associated with ECOG-PS and with the predicted survival outcomes in nm-ccRCC.

Variable importance analysis constitutes another key analytical strength of BNs, enabling the ranking of candidate prognostic variables by their contribution to predictive information. According to Bayes’ theorem, we ranked the top ten prognostic factors based on their relative importance. Our findings demonstrated that the significance of risk factors varied across different time points. Specifically, the ISUP grades were identified as the most critical predictor for 3-year DFS. Furthermore, consistent with prior research (22, 36), the T stages were confirmed as the most significant risk factor for predicting 5-year OS, reaffirming the pivotal role of tumor staging in determining long-term survival outcomes. Although variables such as age, symptom severity, and tumor size were not ranked as highly influential factors in the importance hierarchy, the BN analytical framework not only characterizes the conditional dependencies between independent and dependent variables but also enables the identification of relatively minor and often overlooked contributing factors. This approach offers a more comprehensive and nuanced understanding of the multifactorial associations underlying postoperative progression or death in patients with nm-ccRCC, providing clinically actionable insights from a multivariate perspective.

Several established prognostic tools are available for localized RCC, including the UISS, the Leibovich score, the SSIGN score, the GRANT score and the SYSUCC nomogram (5, 10–12, 18, 19). In a previous external evaluation, Xiong et al. (19) reported 5-year OS AUC values of 0.823 for the SYSUCC nomogram, 0.772 for the Karakiewicz nomogram, 0.792 for the SSIGN prognostic score and 0.701 for pathological staging in 857 patients with non-metastatic RCC treated by radical nephrectomy. Our BN models achieved a 5-year OS AUC of 0.878 in the internal validation cohort and 0.838 in the external validation cohort, together with 3-year DFS AUC values of 0.867 and 0.846, respectively. However, AUC values obtained in different studies are not directly comparable, because patient populations, stage distributions, endpoint definitions, follow-up duration, event rates and validation strategies differ substantially. These differences preclude any claim of superiority over existing models, and we therefore interpret our results as indicating favorable, but not demonstrably superior, discriminative performance; a head-to-head comparison within a common validation cohort would be required to establish any comparative advantage, and the modest size and single-country setting of our cohorts further limit the strength of any comparative claim. To contextualize the model output against reported outcomes, the observed 5-year OS and 3-year DFS rates in the external validation cohort (85.9% and 80.6%, respectively) were concordant with published 5-year OS estimates of approximately 78-92% and 3-year recurrence-free estimates of approximately 74-90% reported for patients with localized RCC (5, 19), and the calibration intercepts and slopes reported above indicate that the predicted probabilities themselves, and not only the ranking of patients, are reliable within this range.

Notwithstanding these promising results, it is imperative to acknowledge the limitations intrinsic to this study. The performance and generalizability of machine learning models are critically contingent upon the robustness, quality, and volume of the training dataset. This study was a retrospective analysis with training data confined to two centers, which may limit the generalizability of the findings. To augment the representativeness of the study cohort and corroborate the external validity of the models, it is essential for future research endeavors to engage in cross-regional, multicenter, large-scale prospective studies. Furthermore, the current set of variables is limited, lacking the integration of cutting-edge parameters such as molecular biomarkers and circulating tumor DNA. Subsequent research initiatives should aim to prospectively amass a more extensive array of variable data, thereby enhancing the model’s comprehensiveness and clinical utility. In addition, a 5-year DFS endpoint was not modeled, because progression status beyond 3 years after surgery could not be reliably ascertained for all patients, as set out above; a 5-year DFS model will be developed and reported in a future prospective, multi-institutional study with a protocol-defined uniform surveillance schedule extending beyond 3 years. Finally, because the clinically decisive objective of postoperative risk stratification is to identify patients at high risk of recurrence or death, clinical translation of these models will require the decision threshold to be re-tuned toward higher sensitivity for the adverse-event class, accepting some reduction in specificity; the performance reported above corresponds to the default operating point defined in the Methods.

## Conclusion

This study establishes BNs for the first time as a clinically actionable tool for postoperative risk prediction in nm-ccRCC, providing individualized 5-year OS and 3-year DFS estimates through the dynamic integration of nonlinear variable interactions. Across the internal and external validation cohorts, the models demonstrated promising discriminative and calibrated performance, supporting their potential application in patient counseling, risk-stratified surveillance, and the selection of candidates for adjuvant therapy or clinical trials. Future prospective, multicenter studies with protocol-defined imaging follow-up are warranted to extend these predictions to a 5-year DFS endpoint and to refine the decision threshold for clinical translation.

## Data Availability

The data analyzed in this study is subject to the following licenses/restrictions: access to the dataset is restricted due to patient privacy protection and hospital ethical and data management regulations. The original clinical medical records involve sensitive personal health information, and data de-identification cannot completely eliminate potential re-identification risks. Therefore, the dataset cannot be publicly shared or made openly available. Qualified researchers may apply for data access upon reasonable request after institutional ethical approval. Requests to access these datasets should be directed to Shushang Chen, fjzlcss@126.com.
