# Supplementary material for: Analysis of prognostic factors for survival after curative resections of clear-cell renal cell carcinoma based on Bayesian networks
Source: Front Oncol. 2026 Oct 28;16:1877632. doi: 10.3389/fonc.2026.1877632 (PMC13751446; doi:10.3389/fonc.2026.1877632)
Supplement: Supplementary file 1 [file Table1.doc]

**TABLE 1** Univariate Cox regression model predicting OS based on the training cohort

| Variable | HR | 95% CI | P |
| --- | --- | --- | --- |
| Age(year) |  |  |  |
| ≤ 50 |  |  |  |
| 51- 69 | 1.485 | 0.724-3.049 | 0.281 |
| ≥ 70 | 4.936 | 2.159-11.283 | <0.001 |
| Sex |  |  |  |
| Male |  |  |  |
| Female | 0.710 | 0.379-1.332 | 0.286 |
| BMI(kg/m²) |  |  |  |
| < 18.5 |  |  |  |
| 18.5-24.9 | 0.774 | 0.238-2.512 | 0.670 |
| ≥ 25 | 0.529 | 0.149-1.876 | 0.324 |
| Smoking history |  |  |  |
| No |  |  |  |
| Yes | 0.882 | 0.494-1.575 | 0.671 |
| Diabetes |  |  |  |
| No |  |  |  |
| Yes | 0.912 | 0.411-2.024 | 0.821 |
| Hypertension |  |  |  |
| No |  |  |  |
| Yes | 1.258 | 0.705-2.246 | 0.437 |
| Heart diseases |  |  |  |
| No |  |  |  |
| Yes | 1.884 | 0.678-5.237 | 0.224 |
| Symptoms |  |  |  |
| No |  |  |  |
| Yes | 3.089 | 1.783-5.352 | <0.001 |
| ECOG-PS |  |  |  |
| 0 |  |  |  |
| ≥ 1 | 4.521 | 2.597-7.872 | <0.001 |
| Laterality |  |  |  |
| left |  |  |  |
| right | 0.988 | 0.573-1.706 | 0.966 |
| Serum albumin |  |  |  |
| < 35 g/L |  |  |  |
| ≥ 35 g/L | 0.651 | 0.306-1.384 | 0.265 |
| eGFR(mL/min/1.73m^2^) |  |  |  |
| ≥ 90 |  |  |  |
| 60-89 | 1.877 | 0.889-3.965 | 0.099 |
| < 59 | 3.778 | 1.767-8.076 | 0.001 |
| ISUP grades |  |  |  |
| 1 |  |  |  |
| 2 | 3.599 | 0.812-15.947 | 0.092 |
| 3 | 13.463 | 3.180-56.994 | <0.001 |
| 4 | 63.343 | 14.210-282.365 | <0.001 |
| Type of surgery |  |  |  |
| PN |  |  |  |
| RN | 2.318 | 1.323-4.062 | 0.003 |
| Tumor necrosis |  |  |  |
| No |  |  |  |
| Yes | 5.174 | 2.895-9.248 | <0.001 |
| Microvascular invasion |  |  |  |
| No |  |  |  |
| Yes | 16.185 | 8.935-29.317 | <0.001 |
| T stages |  |  |  |
| T1a |  |  |  |
| T1b | 1.916 | 0.695-5.284 | 0.209 |
| T2 | 6.049 | 2.191-16.703 | 0.001 |
| T3a | 21.346 | 9.017-50.532 | <0.001 |
| T3b-T4 | 19.459 | 7.041-53.783 | <0.001 |
| N stages |  |  |  |
| N0 |  |  |  |
| N1 | 7.857 | 4.165-14.820 | <0.001 |
| Maximal tumor diameter(cm) |  |  |  |
| ≤ 4 |  |  |  |
| > 4, ≤ 7 | 4.165 | 1.943-8.931 | <0.001 |
| > 7 | 6.888 | 3.088-15.366 | <0.001 |

*BMI* Body Mass Index, *ECOG-PS* Eastern Cooperative Oncology Group Performance Status, *eGFR* Estimated Glomerular Filtration Rate, *PN* Partial nephrectomy, *RN* Radical nephrectomy, *ISUP* International Society of Urological Pathology

**TABLE 2** Univariate Cox regression model predicting DFS based on the training cohort

| Variable | HR | 95% CI | P |
| --- | --- | --- | --- |
| Age(year) |  |  |  |
| ≤ 50 |  |  |  |
| 51- 69 | 1.416 | 0.767-2.614 | 0.266 |
| ≥ 70 | 3.101 | 1.407-6.836 | 0.005 |
| Sex |  |  |  |
| Male |  |  |  |
| Female | 0.820 | 0.470-1.430 | 0.484 |
| BMI(kg/m²) |  |  |  |
| < 18.5 |  |  |  |
| 18.5-24.9 | 0.955 | 0.296-3.077 | 0.938 |
| ≥ 25 | 0.787 | 0.231-2.686 | 0.702 |
| Smoking history |  |  |  |
| No |  |  |  |
| Yes | 1.106 | 0.665-1.839 | 0.698 |
| Diabetes |  |  |  |
| No |  |  |  |
| Yes | 0.869 | 0.414-1.824 | 0.710 |
| Hypertension |  |  |  |
| No |  |  |  |
| Yes | 1.115 | 0.651-1.910 | 0.691 |
| Heart diseases |  |  |  |
| No |  |  |  |
| Yes | 1.982 | 0.795-4.943 | 0.142 |
| Symptoms |  |  |  |
| No |  |  |  |
| Yes | 3.104 | 1.894-5.089 | <0.001 |
| ECOG-PS |  |  |  |
| 0 |  |  |  |
| ≥ 1 | 4.634 | 2.803-7.659 | <0.001 |
| Laterality |  |  |  |
| left |  |  |  |
| right | 0.938 | 0.572-1.538 | 0.800 |
| Serum albumin |  |  |  |
| < 35 g/L |  |  |  |
| ≥ 35 g/L | 0.720 | 0.355-1.458 | 0.361 |
| eGFR(mL/min/1.73m^2^) |  |  |  |
| ≥ 90 |  |  |  |
| 60-89 | 2.195 | 1.127-4.273 | 0.021 |
| < 59 | 3.306 | 1.616-6.766 | 0.001 |
| ISUP grades |  |  |  |
| 1 |  |  |  |
| 2 | 2.470 | 0.704-8.669 | 0.158 |
| 3 | 12.668 | 3.871-41.459 | <0.001 |
| 4 | 65.130 | 18.793-225.713 | <0.001 |
| Type of surgery |  |  |  |
| PN |  |  |  |
| RN | 3.111 | 1.830-5.288 | <0.001 |
| Tumor necrosis |  |  |  |
| No |  |  |  |
| Yes | 6.561 | 3.916-10.995 | <0.001 |
| Microvascular invasion |  |  |  |
| No |  |  |  |
| Yes | 26.116 | 14.879-45.839 | <0.001 |
| T stages |  |  |  |
| T1a |  |  |  |
| T1b | 13.595 | 3.126-59.130 | 0.001 |
| T2 | 28.855 | 6.394-130.218 | <0.001 |
| T3a | 98.660 | 23.356-416.755 | <0.001 |
| T3b-T4 | 76.308 | 16.175-360.002 | <0.001 |
| N stages |  |  |  |
| N0 |  |  |  |
| N1 | 13.917 | 7.977-24.281 | <0.001 |
| Maximal tumor diameter(cm) |  |  |  |
| ≤ 4 |  |  |  |
| > 4, ≤ 7 | 12.927 | 4.586-36.438 | <0.001 |
| > 7 | 21.737 | 7.559-62.506 | <0.001 |

*BMI* Body Mass Index, *ECOG-PS* Eastern Cooperative Oncology Group Performance Status, *eGFR* Estimated Glomerular Filtration Rate, *PN* Partial nephrectomy, *RN* Radical nephrectomy, *ISUP* International Society of Urological Pathology

**TABLE 3** The conditional probability of T stages according to the BN model for 5-year OS

| Parent node | | Conditional probability of T stages | | | | |
| --- | --- | --- | --- | --- | --- | --- |
| eGFR  (mL/min/1.73m2) | 5-year OS | T1a | T1b | T2 | T3a | T3b-T4 |
| ≥ 90 | Yes | 0.63 | 0.31 | 0.04 | 0.02 | 0.01 |
| ≥ 90 | NO | 0.01 | 0.15 | 0.15 | 0.69 | 0.01 |
| 60-89 | Yes | 0.49 | 0.35 | 0.10 | 0.06 | <0.01 |
| 60-89 | NO | 0.06 | 0.21 | 0.16 | 0.42 | 0.16 |
| < 59 | Yes | 0.36 | 0.23 | 0.21 | 0.12 | 0.08 |
| < 59 | NO | <0.01 | <0.01 | 0.19 | 0.49 | 0.31 |

**TABLE 4** The conditional probability of ISUP grades according to the BN model for 3-year DFS

| Parent node | | Conditional probability of ISUP grades | | | |
| --- | --- | --- | --- | --- | --- |
| N stages | 3-year DFS | 1 | 2 | 3 | 4 |
| N0 | Yes | 0.29 | 0.51 | 0.19 | 0.01 |
| N0 | NO | <0.01 | 0.20 | 0.57 | 0.23 |
| N1 | Yes | 0.03 | 0.03 | 0.92 | 0.03 |
| N1 | NO | 0.06 | 0.11 | 0.44 | 0.39 |
